# Mind the Gap: Genetic Manipulation of Basicranial Growth within Synchondroses Modulates Calvarial and Facial Shape in Mice through Epigenetic Interactions

**DOI:** 10.1371/journal.pone.0118355

**Published:** 2015-02-18

**Authors:** Trish E. Parsons, Charlene M. Downey, Frank R. Jirik, Benedikt Hallgrimsson, Heather A. Jamniczky

**Affiliations:** 1 McCaig Institute for Bone and Joint Health, University of Calgary, Calgary, Canada; 2 Department of Cell Biology & Anatomy, Cumming School of Medicine, University of Calgary, Calgary, Canada; 3 Department of Biochemistry and Molecular Biology, Cumming School of Medicine, University of Calgary, Calgary, Canada; University of Liverpool, UNITED KINGDOM

## Abstract

Phenotypic integration patterns in the mammalian skull have long been a focus of intense interest as a result of their suspected influence on the trajectory of hominid evolution. Here we test the hypothesis that perturbation of cartilage growth, which directly affects only the chondrocranium during development, will produce coordinated shape changes in the adult calvarium and face regardless of mechanism. Using two murine models of cartilage undergrowth that target two very different mechanisms, we show that strong reduction in cartilage growth produces a short, wide, and more flexed cranial base. This in turn produces a short, wide face in both models. Cranial base and face are already correlated early in ontogeny, and the relationship between these modules gains structure through postnatal growth and development. These results provide further evidence that there exist physical interactions between developing parts of the phenotype that produce variation at a distance from the actual locus upon which a particular selective pressure is acting. Phenotypic changes observed over the course of evolution may not all require adaptationist explanations; rather, it is likely that a substantial portion of observed phenotypic variation over the history of a clade is not directly adaptive but rather a secondary consequence of some local response to selection.

## Introduction

Previous studies of integration patterns in the mammalian skull have determined that it is comprised of three modules: the basicranium, the calvarium, and the face [1,2]. These individual modules are of distinct embryonic origin and undergo varying modes of growth [3]. They have been shown to interact molecularly and physically throughout development, however, and form a single, multifunctional, integrated unit [4–6]. Considerable effort has been invested in determining the precise relationships between these modules, and their roles in structuring much of the phenotypic variation that has influenced the trajectory of primate, and hominid, craniofacial evolution [7–11].

The basicranium is of particular interest to evolutionary developmental biologists: firstly, it is centrally located in the skull, above the face and below the calvarium, and provides the platform upon which the brain rests; secondly, it reaches adult size prior to the calvarium and face, and thus sets the parameters for the rest of the skull as it grows [12,13]; thirdly, the basicranium grows via endochondral ossification within its synchondroses, making its shape and growth more likely to be under intrinsic control [14]. The basicranium differs in this respect from the calvarium and face, which are regulated by brain expansion [15] and growth hormone expression [16], respectively. The basicranium is widely thought to be central to growth and patterning of the skull, and by extension the skull’s evolutionary trajectory in mammals [3,8,12,17,18]. Growth of the basicranium by expansion from within the synchondroses is likely key to this role.

Developmentally determined biases in the generation of phenotypic variation can influence the multivariate direction of evolutionary change by altering patterns of integration among traits [19,20]. Integration patterns are produced when variation in the underlying developmental architecture affects various parts of the organism unequally, resulting in correlated variation among related traits [2,4]. Correlated variation of this nature is often mediated by epigenetic effects on developing parts of a complex phenotype, where physical interactions among tissues reciprocally influence adult morphology [6,21–24]. This underlying architecture is itself a product of evolution, and the correlated variation it produces will in turn enhance the evolvability of such complex developmental systems through coordinated responses to selection [25,26].

The complex relationship between developmental architecture and natural selection makes the determination of the precise locus of selection challenging. The intricate nature of these connections among different aspects of the phenotype supports the notion that considerable phenotypic variation present during the evolutionary history of a clade may not necessarily arise directly from the action of natural selection on those phenotypes; rather, much of this variation may arise incidentally as the result of developmental architecture [20,27,28]. Recent work, however, has begun to reveal that the role of developmental architecture in modulating evolution has been vastly underappreciated, and that investigating evolution through a focus on developmental architecture allows novel hypotheses about the means by which different characters have evolved to be tested in a variety of settings [2,29–35].

Here, we take such a ‘middle-out’ approach to the evolutionary history of the mammalian skull by targeting high-level developmental processes to investigate phenotypic effects [31]. We test the hypothesis that perturbation of the unique developmental process responsible for basicranial growth, namely expansion within synchondroses, will result in coordinated changes in the calvarium and face even though the latter two structures do not employ this unique process. We use two murine models of cartilage ‘undergrowth’ and one of cartilage ‘overgrowth’, perturbing the growth of the basicranium via three very different mechanisms, and examine shape and phenotypic covariance outcomes in neonatal and adult skulls. This experimental design allows us to assess both the effects of different mechanisms on skull components, as well as the relationships between skull components and changes in the patterns of these relationships across developmental time.

## Materials and Methods

### Sample Composition

Two transgenic mouse strains were employed in this study, both of which are products of Cre-*lox*P technology targeting specific alleles in tissues expressing the Col2a1-Cre transgene (Jackson Laboratory, Bar Harbour, ME) which directs osteo-chondroprogenitor-specific gene alterations. *Pten* ^*fl/fl*^ Col2a1Cre mice are a transgenic strain developed on a C57BL/6 background, that have *Pten* deletions in osteo-chondroprogenitor cells [36]. *Pten* is a tumor suppressor gene that negatively regulates the phosphatidylinositol 3’-kinase signaling pathway (PI3K) [37]. This pathway plays an important role in regulating both normal cartilage and subsequent endochondral bone development. The deletion of *Pten* leads to over-activation of the PI3K pathway, and subsequently produces endochondral bone overgrowth [36]. As the chondrocranium is the only completely endochondral module of the skull, this module is the only one directly affected by the *Pten* deletion. We employ these mice as the ‘overgrowth’ model (OG). Unaffected littermates that did not express the Cre transgene were used as controls for this group (OGc). Cre activity in the OG sample was confirmed using β-galactosidase staining and PCR as described in [36].

*Trsp* ^*fl/fl*^ Col2a1-Cre mice are a transgenic strain developed on a C57BL/6J background that have deletions of the *Trsp* gene in osteo-chondroprogenitor cells [38]. *Trsp* encodes a transfer RNA, which is responsible for the insertion of the selenocysteine residues into polypeptides encoded by the selenoprotein gene family. Among their many roles, selenoproteins function as antioxidants, protecting cells against damage from oxidative stress [39]. Mice that lack a functional *Trsp* gene, and therefore do not express selenoproteins in cartilaginous tissue, exhibit retarded growth, abnormal epiphyseal growth plates, delayed endochondral ossification, and chondronecrosis [38]. We employ these mice as an ‘undergrowth’ model (UG1). Unaffected littermates that did not express the Cre transgene were used as controls for this group (UG1c). Cre activity in the UG1 sample was confirmed using PCR as described in [38].

‘Brachymorph’ mice exhibit an autosomal recessive mutation in the phosphoadenosine-phosphosulfate synthetase gene (*Papps2*) [40]. This mutation causes a substantial decrease in sulfation of glycosaminoglycans, which results in proteoglycan groups that are both small and reduced in number in the extracellular matrix [41]. Cartilage growth is drastically reduced and all endochondral bones are abnormally small as a morphological consequence. As in the transgenic mice described above, only the chondrocranium is affected; the *Papps2* gene does not control or mediate development in any way [42]. We employ these mice as a second adult ‘undergrowth’ model (UG2). The brachymorph population used here was developed on a C57BL6/J genetic background, and therefore C57BL6/J mice were used as controls for this group (UG2c). Neonatal skulls were not available for this group and are not included here.

### Data Collection, Scanning and 3D Reconstruction

Neonatal mice of indeterminate sex from both transgenic strains (OG n = 12; OGc n = 19; UG1 n = 19; UG1c n = 19; total n = 69) were collected on postnatal day (P) 2 and sacrificed via intracardiac injection of 0.25–0.5 mL Euthanyl (240mg mL-1 sodium pentobarbital) while under isoflurane anaesthesia. Specimens were kept frozen until the time of micro-computed tomography (μCT) scanning. Crania were scanned in situ using a Scanco μCT40 (SCANCO Medical AG, Brüttisellen, Switzerland) at a standard setting of 45 kVp, 109 μA, with an isometric voxel size of 0.12 mm. Reconstructed μCT slices were converted to 3D surface renderings using a standardized threshold value for neonatal bone, and these surfaces were used for further analysis (Amira 5.0, Visage Imaging, Carlsbad, CA, USA).

All of the adult samples used for this study were included in previous studies (OG n = 24; OGc n = 18; UG1 n = 10; UG1c n = 9; UG2 n = 30; UG2c n = 20) [4,20,42]. The OG sample was originally obtained from Jackson Laboratories (Bar Harbor, ME, USA), while the transgenic mice were generated at the University of Calgary as described previously [36,38]. The UG1 sample was limited in size due to the detrimental effect of the gene deletion on cartilage, which eventually led to deteriorating health including increasing difficulty in breathing, leading to high mortality beyond five weeks of age [38]. Following euthanasia, crania were stored at -20°C. Crania were μCT scanned with a Scanco vivaCT40 (SCANCO Medical AG, Brüttisellen, Switzerland) at 55kVp, 109 μA, with an isometric voxel size of 0.35 mm. Reconstructed μCT slices were converted to 3D surface renderings using a standardized threshold value for adult bone, and these surfaces were used for further analysis (Amira 5.0, Visage Imaging, Carlsbad, CA, USA).

Ethics Statement. All mice were housed, and later sacrificed via CO_2_ inhalation in the case of adult specimens and via intracardiac injection of Euthanyl while under isoflurane anaesthesia in the case of neonates, following an approved protocol and the guidelines of the University of Calgary Animal Care Committee and the Canadian Council on Animal Care.

### Landmarking

**Fig 1:**
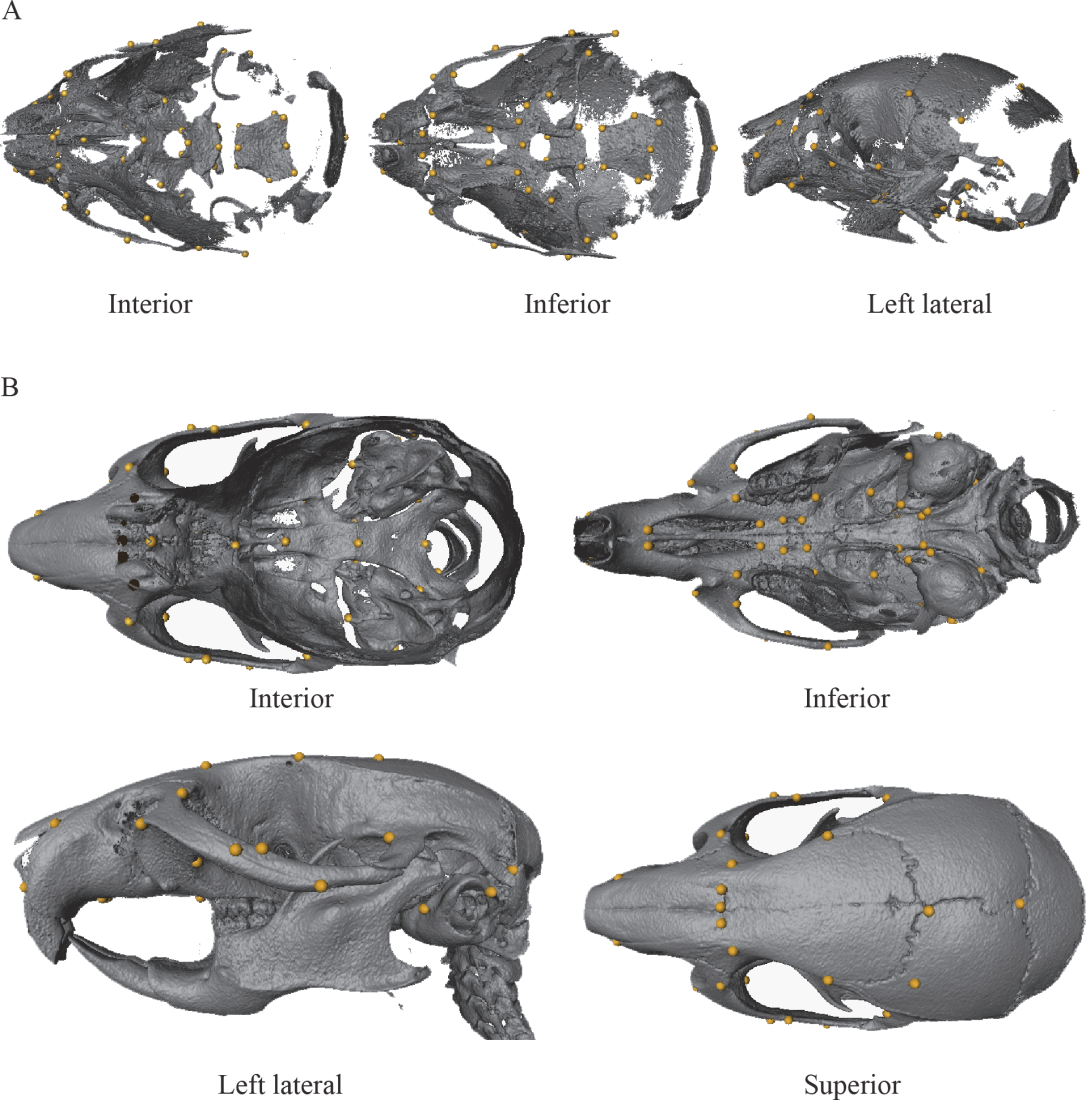
Neonatal (A) and adult (B) landmarks used in this study. Landmark descriptions and numbers are provided in Table 1.

Overlapping subsets of a global landmark set (Fig. 1; Table 1) were collected on both neonates and adults. Adult volume renderings were landmarked with Analyze 5.0 (Mayo Foundation for Medical Education and Research, Rochester, MN, USA) while neonatal renderings were landmarked using Amira 5.0 (Visage Imaging, Carlsbad, CA, USA). The final datasets included 58 landmarks for the neonatal analysis and 59 for the adult.

**Table 1:** Landmark information including: descriptions, whether they were taken on adults or neonates or both, and their assigned modules.

**LM**	**Description**	**Age**	**Module**
M1	Midline superior incisor	B	Fa
R/L 2	Anterior margin of palatine foramen	A	Fa
R/L 3	Anterior point on the inferior zygomatic process	B	Fa
R/L 4	Point of greatest curvature on the posterior malar process	B	na
R/L 5	Anterior point on the superior alveoli	B	Fa
R/L 6	Posterior point on palatine foramen	A	Fa
R/L 7	Point of the palatine-maxillary suture	A	Fa
R/L 8	Posterior point of the superior alveoli	B	Fa
R/L 9	Lateral palatal pterygoid junction	N	Ba
R/L 10	Spheno-occipital synchondrosis	B	Ba
R/L 11	Anterior point of the foramen ovale	B	Ba
R/L 12	Anterior point of the inferior auditory bulla	A	Ba
R/L 13	Point of greatest curvature along the posterior zygomatic process	A	Ba
R/L 14	Occipital-auditory-sphenoid junction	B	Ba
R/L 15	Occipital-auditory bulla junction	B	Ba
R/L 16	Posterior point of the basioccipital	N	Ba
R/L 17	Auditory-temporal-sphenoid junction	B	Ba
R/L 18	Medial palatal-pterygoid junction	B	Ba*
R/L 19	Medial maxilllary-premaxilla junction	A	Fa
R/L 20	Anterior-most point along the lateral zygomatic-frontal suture	N	Fa
M 21	Nasion	B	Fa
R/L 22	Lateral point along frontal-maxilla suture	B	Ca
R/L 23	Intersection of frontal suture with orbital arm	B	Ca
R/L 24	Superior margin of temporal-zygomatic suture	B	Fa
R/L 25	Frontal-temporal-parietal junction	B	Ca
R/L 26	Bregma	B^	Ca
R/L 27	Lamda	B^	Ca
R/L 28	Point along occipitomastoid suture	B	Ca
R/L 29	Superioposterior tympanic ring	B	Ca
R/L 32	Posterior point of the temporal-zygomatic junction	A	na
R/L 33	Anterior point of the temporal-zygomatic junction	A	na
R/L 34	Posterior zygomatic-frontal junction	A	Ca
R/L 35	Anterior point of the nasal-premaxilla suture	B	Fa
M 36	Basion	B	Ba
M 37	Midline occipital basisphenoid synchondrosis	B	Ba
M 38	Midline basisphenoid-presphenoid synchondrosis	B	Ba
M 39	Presphenoid-palatine suture	B	Ba
M 40	Foramen caecum	B	Ca
M 41	Subspinale	B	Fa
M42	Euryon; on parietal at ends of greatest transverse diameter of skull	N	Ca
M45	Hormion; point of union of sphenoid and vomer	N	na

Abbreviations: LM = landmark number; Abbr = abbreviation; A = Adult; B = both; N = Neonate; Fa = Face; Ba = Basicranium; Ca = calvarium; na = not included in a module

* = adult analysis only, ^ = landmark for neonate is not yet ossified, and so was taken on soft tissue in the area surrounded by developing calvarial bones: location ‘bregma’ surrounded by frontal and parietal, and location ‘lambda’ surrounded by parietal and interparietal.

### Shape Analyses

Raw landmark coordinates were Procrustes-transformed to remove the effects of scaling, rotation and translation. Allometric information was retained in the shape data for these analyses (see Discussion for a justification of this decision). Principal component analysis (PCA) was performed on the Procrustes-transformed coordinates to visualize patterns of variation in each dataset. PCA is an ordination technique that reduces the dimensionality of a large dataset, and provides a means of visualizing the greatest axes of shape variation among individuals in that dataset. Procrustes distances between group means were calculated in a pairwise fashion, but given the small sample sizes in each of our treatment groups we did not extend these calculations to a canonical variates analysis [43].

### Covariation Analyses

Landmarks were assigned to three subsets (Table 1) in order to test for covariation between the different modules of the skull: face, calvarium, and basicranium. When using discrete landmarks, some aspects of the murine skull are sampled more heavily than others (e.g. the face versus the calvarium). An effort was made towards an equitable distribution of landmarks in each module. Additionally, in many cases there are landmarks that border two modules. Assigning them to one module or the other could skew the results; however, these decisions had to be made because, in many cases, to disregard those landmarks altogether would lead to missing information from an entire area. As a result, some landmarks were not assigned to any module. Group-mean centered two-block partial least squares (PLS) analyses were used to test for covariation between these modules [44,45], with each module being considered a separate block rather than parts of the same configuration. A group-mean centred approach was chosen to best account for between- and within-group variation. In this analysis, each landmark subset underwent an independent Procrustes fit. Although the modules do exist in one structure, and a PLS within a single Procrustes configuration would provide covariation information in the context of the entire skull, it could possibly inflate the amount of covariation between the two modules [46]. We have chosen the more conservative approach here. Percent total variation explained by each PLS axis was calculated by regressing PLS scores on the original data.

All geometric morphometric analyses were performed in MorphoJ v 1.05f [47]. All statistical significance tests were computed via nonparametric permutation tests with 10000 permutations. Skull morphs were constructed using Landmark [48].

## Results

### Cartilage undergrowth produces shortened basicrania in mice across ontogeny

Procrustes distances between mean skull shapes, which represent how different mean shapes are between groups, are reported in Tables 2 and 3. The greatest distance between any two neonatal groups is between the OG and UG1 mice. The greatest distance between any two adult groups is between the OGc and UG2 mice. In all cases, the mean skull shapes of all groups were significantly different from each other.

**Table 2:** Procrustes distances between neonatal mean shapes.

	**OGc**	**OG**	**UG1c**
OG	0.0332		
UG1c	0.0295	0.0332	
UG1	0.0330	0.0427	0.0231

All comparisons are significant at p < 0.0001 except UG1 -> UG1c, which is significant at p = 0.0102. Abbreviations: OG = Pten overgrowth model; OGc = overgrowth control, UG1 = Trsp undergrowth model; UG1c = Trsp undergrowth control. See Materials and Methods for a full explanation of these models.

**Table 3:** Procrustes distances between adult mean shapes.

	**UG2**	**UG2c**	**OG**	**OGc**	**UG1**
UG2c	0.0886				
OG	0.0916	0.0487			
OGc	0.0959	0.0353	0.0409		
UG1	0.0593	0.1058	0.1085	0.1163	
UG1c	0.0783	0.0436	0.0545	0.0512	0.0783

All comparisons are significant at p < 0.0001. Abbreviations: OG = Pten overgrowth model; OGc = overgrowth control, UG1 = Trsp undergrowth model; UG1c = Trsp undergrowth control; UG2 = Brachymorph undergrowth model; UG2c = Brachymorph undergrowth control (C57BL6/J). See Materials and Methods for a full explanation of these models.

**Fig 2:**
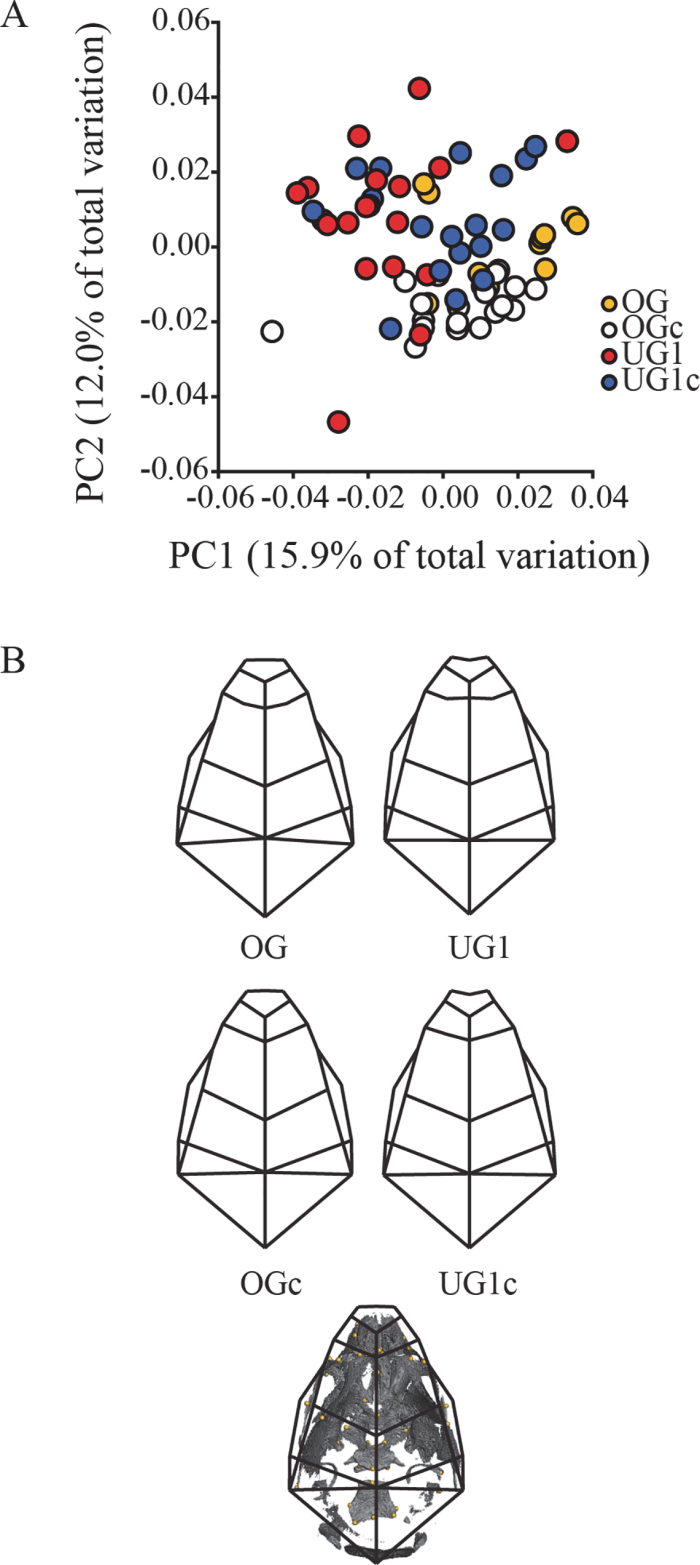
Principal components analysis (PCA) of neonatal mouse skulls. A) Scatter plot of PC1 and PC2 of neonatal whole-skull shape. B) Wireframe depictions of group mean shapes. Abbreviations: PC = principal component; OG = Pten overgrowth model; OGc = overgrowth control, UG1 = Trsp undergrowth model; UG1c = Trsp undergrowth control. See Materials and Methods for a full explanation of these models.

A biplot of the first two principal components (PC) from the neonatal analysis shows considerable overlap between the four groups (Fig. 2A), and a comparison of group mean shapes reveals that phenotypic differences between groups are very subtle at this stage (Fig. 2B). The UG specimens exhibit a slightly shorter cranial base, reduced facial protrusion and a slightly more domed calvarium in comparison with the OG specimens. Further, the UG specimens exhibit a more rounded face in comparison to their controls, while the OG mice are almost indistinguishable from their controls.

**Fig 3:**
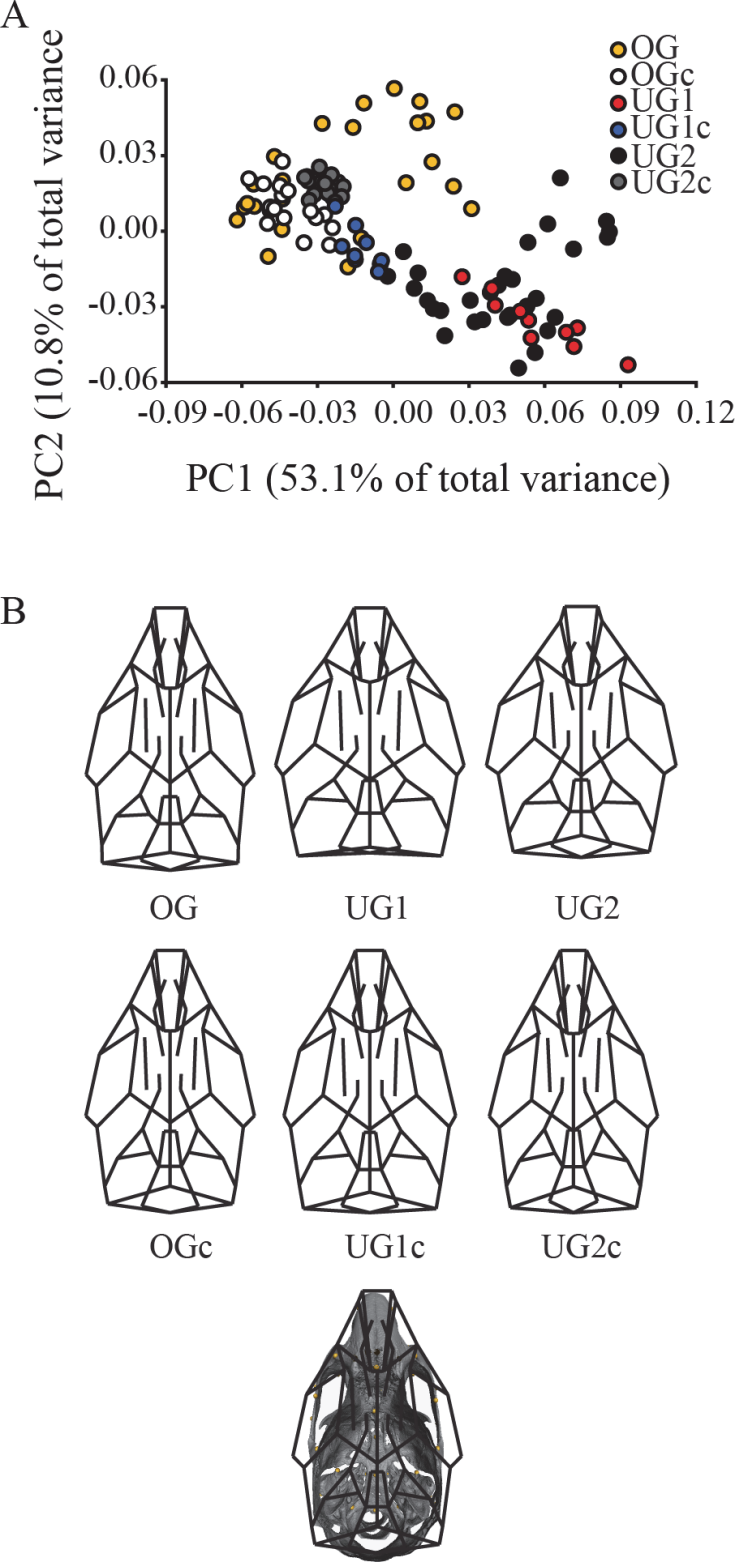
Principal components analysis (PCA) of adult mouse skulls. A) Scatter plot of PC1 and PC2 of adult whole-skull shape. B) Wireframe depictions of group mean shapes. Abbreviations: PC = principal component; OG = Pten overgrowth model; OGc = overgrowth control, UG1 = Trsp undergrowth model; UG1c = Trsp undergrowth control; UG2 = Brachymorph undergrowth model; UG2c = brachymorph undergrowth control. See Materials and Methods for a full explanation of these models.

PCA of the adult dataset reveals relatively stronger group structure (Fig. 3A). UG1 and UG2 mainly occupy the positive end of PC1 with their respective controls. The OG group clusters with its control at the negative end of the plot, but occupies a broader region of morphospace that extends toward the positive end. Comparison of group mean shapes (Fig. 3B) reveals that the UG1 and UG2 mice share a broadly similar skull shape, differing principally from each other in the structure of the posteriormost skull, where UG1 exhibits a relatively shorter vault. UG1 and UG2 differ from OG in a similar fashion, where both undergrowth models exhibit a shorter basicranium and a more domed calvarium, in contrast to the flat calvarium and elongate basicranium of the overgrowth model. The three control groups are broadly similar and cluster together in the biplot (Fig. 3A), occupying a restricted portion of the morphospace and exhibiting substantially less variation than the experimental groups.

### Face and calvarium shape are highly correlated with basicranial shape in neonatal mice

**Fig 4:**
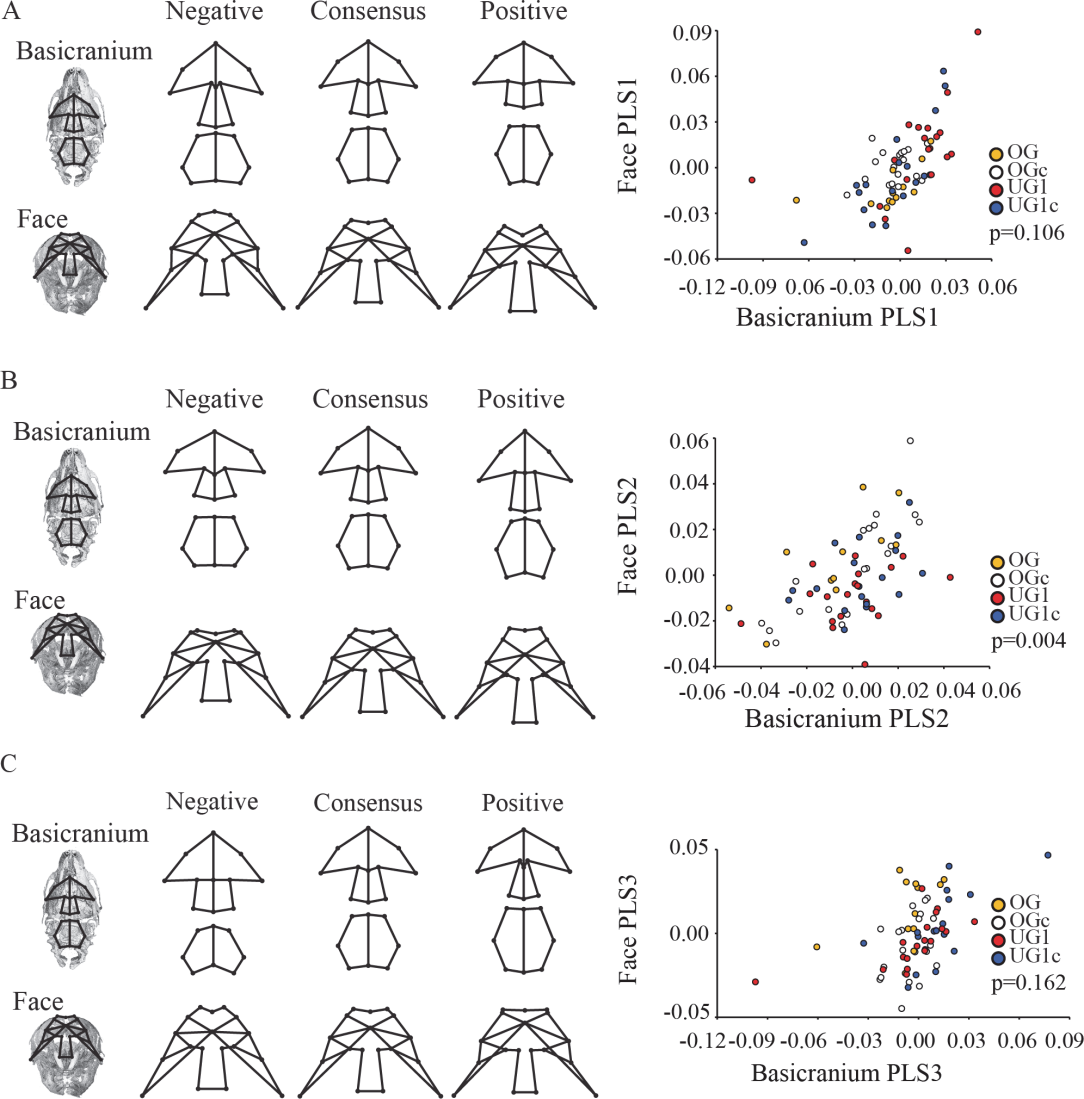
Two-block partial least squares analysis (PLS) of the neonatal mouse basicranium and face. Wireframes depict singular warp deformations of shape along each PLS axis. Abbreviations: PLS = partial least squares axis; OG = Pten overgrowth model; OGc = overgrowth control, UG1 = Trsp undergrowth model; UG1c = Trsp undergrowth control. See Materials and Methods for a full explanation of these models. Wireframe superimposition on skull renderings indicates approximate position of landmarks under consideration.

The neonatal basicranium and face covary significantly (RV = 0.215; p = 0.004), although the structure of this covariation is complex and the RV is relatively low. The first PLS axis captures 38.3% of the total covariation between these modules, but is not significant (p = 0.106) and does not reveal group structure despite a relatively strong correlation value (0.576; Table 4, Fig. 4A). The shapes of different regions of the basicranium show independent trends of covariation along this axis. At the positive end, the anterior region appears relatively shorter and the posterior appears narrowed, and the opposite is true at the negative end. Each of these basicranial shapes is then associated with a different facial shape. At the negative end of PLS1, the broad basioccipital is also associated with a shortened palate, as indicated by landmarks from anterior alveoli to the posterior alveoli. Paradoxically, the sphenoidal region appears longer and is correlated with an increased protrusion of the anterior face.

**Table 4:** Covariation hypothesis testing results for the neonatal sample.

	**Basicranium—Face**	**p**	**cv**	**v**	**Basicranium—Calvarium**	**p**	**cv**	**v**
RV	0.215	0.004	na	B: 60.1 F: 62.0	0.206	0.0003	na	B: 59.3 C: 70.6
PLS1	0.576	0.106	38.3	na	0.595	0.030	36.6	na
PLS2	0.645	0.004	19.7	na	0.531	0.117	30.7	na
PLS3	0.523	0.162	14.5	na	0.644	0.001	10.9	na

Abbreviations: RV = RV value; p = p-value; PLS = PLS axis; cv = percent covariation explained; v = percent variation in complete dataset explained; B = basicranium; F = face; C = calvarium. See Materials and Methods for a full explanation of this analysis.

PLS2 is significant and captures 19.7% of the total covariation between the basicranium and face, and describes relative width along with basicranial length (Table 4; Fig. 4B). The lateral displacement of the sphenoid is correlated with the lateral displacement of the face as shown in both the zygomatic arches and the palate. The positive end of the basicranial shape axis exhibits an elongate sphenoid and basioccipital, which is associated with a longer nasal region and an anteriorly displaced subspinale along with a relatively narrow face.

PLS3 is not significant but again shows a relatively strong correlation of 0.523, represents 14.5% of the total covariation between the basicranium and face (Table 4; Fig. 4C). This axis is associated with shapes that show a correlation between the overall relative lengths of both modules. The shape correspondences at the positive end of PLS3 show that the bony basal elements of both the sphenoid and basioccipital are elongated in the anterior-posterior direction. This correlates with a short, broad face; the palate is anteriorly shifted, but the distance between the anterior alveolar bone and the subspinale is shortened.

**Fig 5:**
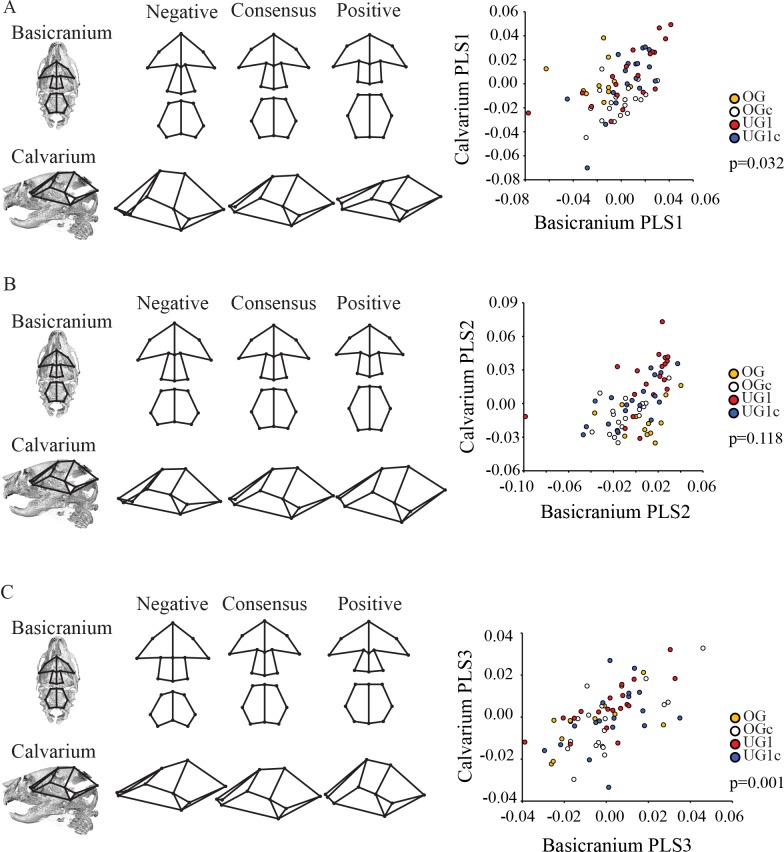
Two-block partial least squares analysis (PLS) of the neonatal mouse basicranium and calvarium. Wireframes depict singular warp deformations of shape along each PLS axis. Abbreviations: PLS = partial least squares axis; OG = Pten overgrowth model; OGc = overgrowth control, UG1 = Trsp undergrowth model; UG1c = Trsp undergrowth control. See Materials and Methods for a full explanation of these models. Wireframe superimposition on skull renderings indicates approximate position of landmarks under consideration.

The neonatal basicranium and calvarium also covary significantly (RV = 0.206; p = 0.003). although there is once again considerable overlap between groups and complex covariation patterns in the context of relatively low RV. The first PLS axis is significant and represents 36.6% of the total covariation (Table 4, Fig. 5A), and again reveals opposing trends in shape between the sphenoid and basioccipital within a configuration. At the positive end of the covariation axis, the sphenoid is relatively short, whereas the basioccipital is antero-posteriorly elongate. This shape is correlated with a calvarial shape that is relatively flatter in both antero-posterior and medio-lateral directions. At the negative end of PLS1, a more robust sphenoid region combined with a more compact basioccipital is associated with a calvarium exhibiting a bregma displaced in the superior direction, which gives the impression of a much more rounded shape.

PLS2, although not significant, represents 30.7% of the total covariation and has relatively strong correlation (0.531; Table 4, Fig. 5B). Inspection of the scatterplot of PLS 2 reveals differences in covariation pattern between the four groups. The OG and OGc groups show almost no correlation, with data points evenly spread along the basicranium axis, while the UG1 and UG1c groups appear to show a correlation between basicranium and calvarium. The positive end of this axis describes a basioccipital that is relatively larger in all directions along with a shorter and smaller sphenoid, which are associated with a longer and narrower calvarium and a higher cranial vault. This suggests that the different bones within the cranial base may covary with different aspects of calvarial shape.

PLS3 is significant and represents 10.9% of the total covariation (Table 4, Fig. 5C). All four groups show the same trend of covariation between the basicranium and calvarium on this axis. Here, a wide cranial base (both sphenoid and basioccipital) is associated with a wide and domed calvarium. A relatively short basioccipital combined with a relatively longer sphenoid are associated with a relatively longer and flatter calvarium.

### Covariation between murine basicranium, face and calvarium becomes more structured across ontogeny

**Fig 6:**
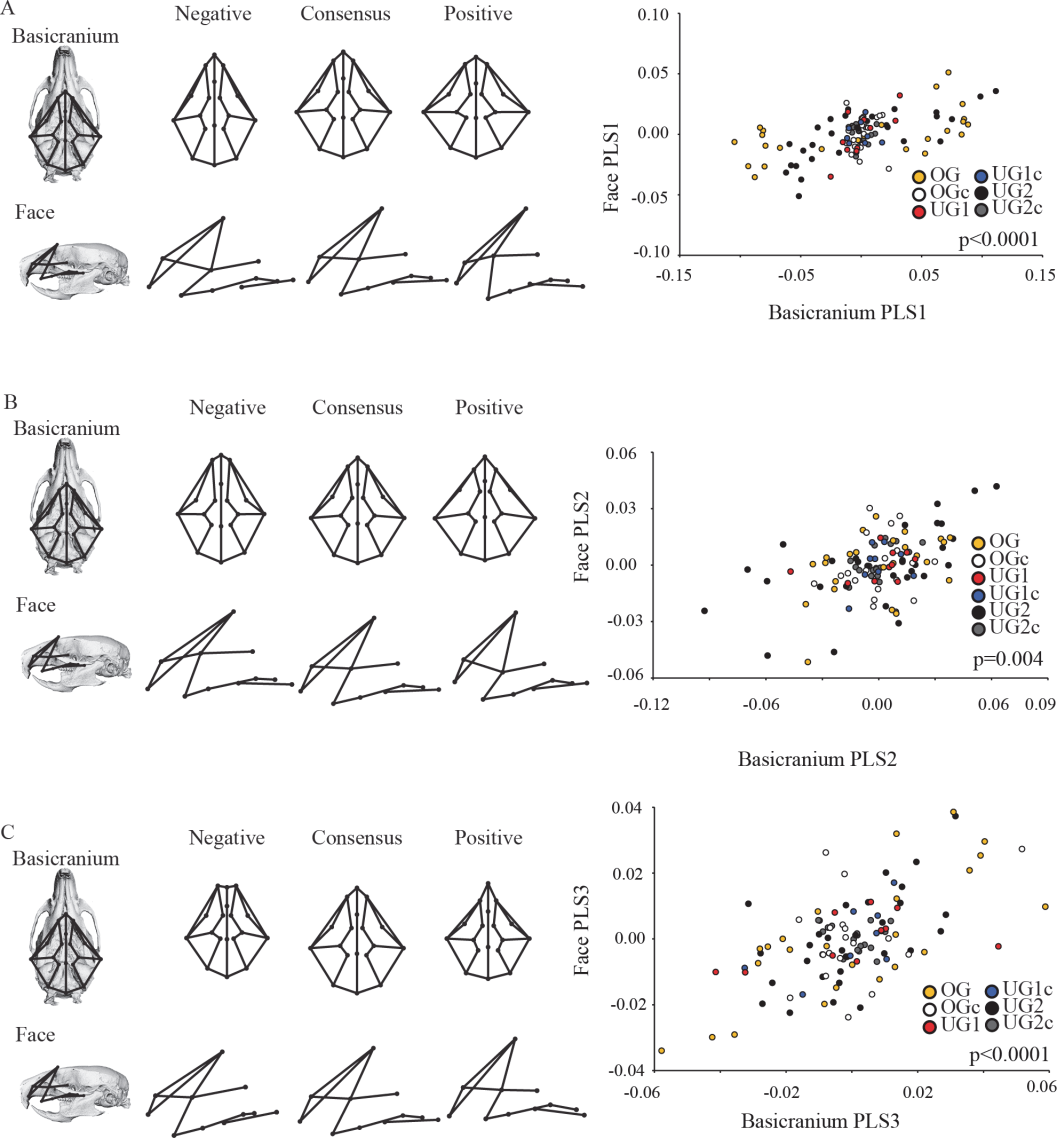
Two-block partial least squares analysis (PLS) of the adult mouse basicranium and face. Abbreviations: PC = principal component; OG = Pten overgrowth model; OGc = overgrowth control, UG1 = Trsp undergrowth model; UG1c = Trsp undergrowth control; UG2 = Brachymorph undergrowth model; UG2c = brachymorph undergrowth control. See Materials and Methods for a full explanation of these models. Wireframe superimposition on skull renderings indicates approximate position of landmarks under consideration.

The adult basicranium and face covary weakly but significantly (RV = 0.136; p <0.0001). PLS1 is significant and represents 63.8% of the total covariation (Table 5, Fig. 6A). The control groups all cluster tightly at the midpoint of the axis while the UG1, UG2 and OG groups display relatively greater variation across the axis, and there is considerable overlap among all groups. The UG1 group displays less variation than the UG2 group. All groups have approximately similar slopes. The positive end of the axis is associated with relatively wider and shorter basicrania, and antero-posteriorly shortened faces. The region of morphospace occupied by the control groups shows relatively narrower and anteriorly pointed basicrania, and relatively longer faces.

**Table 5:** Covariation hypothesis testing results for the adult sample.

	**Basicranium—Face**	**p**	**cv**	**v**	**Basicranium—Calvarium**	**p**	**cv**	**v**
RV	0.136	< 0.0001	na	B: 65.6 F: 64.5	0.650	< 0.0001	na	B: 23.1 C: 64.9
PLS1	0.558	<0.0001	63.8	na	0.903	< 0.0001	90.5	na
PLS2	0.482	0.004	14.2	na	0.696	< 0.0001	5.19	na
PLS3	0.604	<0.0001	9.48	na	0.580	<0.0001	1.65	na

Abbreviations: RV = RV value; p = p-value; PLS = PLS axis; cv = percent covariation explained; v = percent variation in complete dataset explained; B = basicranium; F = face; C = calvarium. See Materials and Methods for a full explanation of this analysis. See Materials and Methods for a full explanation of this analysis.

PLS2 is significant and captures 14.2% of the total covariation (Table 5, Fig. 6B). Once again the control groups cluster together and occupy a relatively restricted portion of morphospace, while the UG1, UG2 and OG groups display considerably more variation, with UG2 substantially more variable than all other groups. Once again, all slopes are similar and there is considerable overlap between groups. This axis reveals an association between breadth of the basicranium and depth of the face in the superior-inferior direction, as well as a rounder basicranium at the positive end of the axis.

PLS 3 is significant and captures 9.48% of the total covariation (Table 5, Fig. 6C). Once again there is considerable overlap among groups, with all groups sharing a similar slope. Similar variation across the axis is present in all groups. The axis shows that a basicranium with a sharply pointed anterior portion is associated with a tall, narrow face while a blunter anterior basicranium is associated with an antero-posteriorly elongate face.

**Fig 7:**
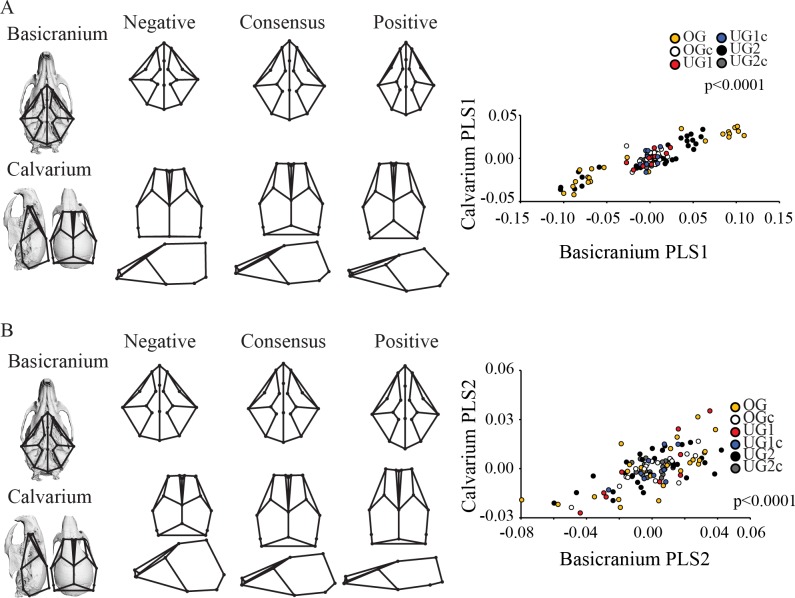
Two-block partial least squares analysis (PLS) of the adult mouse basicranium and calvarium. Abbreviations: PC = principal component; OG = Pten overgrowth model; OGc = overgrowth control, UG1 = Trsp undergrowth model; UG1c = Trsp undergrowth control; UG2 = Brachymorph undergrowth model; UG2c = brachymorph undergrowth control. See Materials and Methods for a full explanation of these models. Wireframe superimposition on skull renderings indicates approximate position of landmarks under consideration.

The calvarium covaries strongly and significantly with the basicranium (RV = 0.650; p < 0.0001). PLS1 and PLS2 account for 95.6% of the covariation in the sample, so only these two axes are discussed below. PLS1 is significant and represents 90.5% of the total covariation (Table 5, Fig. 7A). Basicranial shape changes from relatively long and narrow at the positive end to relatively short and wide at the negative end. Here, the OG and UG2 groups exhibit considerably greater variation than the other groups, and the OG group is divided into two subgroups that cluster at either end of the axis. Control groups occupy a restricted region of morphospace. All groups share a similar slope. Calvarial shape follows a similar trend from positive to negative. At the negative end, the calvarium is relatively shorter, wider and exhibits a higher and more globular shape.

PLS2 is significant and captures 5.2% of the total covariation (Table 5, Fig. 7B), and there is substantial overlap between groups on this axis, with all groups sharing a similar slope and distribution across the axis. The negative end of this axis is characterized by a shortened anterior cranial base and slightly longer posterior cranial base, correlated with a higher and more domed cranial vault. This axis also shows a correlation in relative widths between the cranial base and calvarium.

## Discussion

Many researchers have posited that the cranial base patterns the rest of the skull, and changes to its shape could have cascading consequences [7–11,49]. It is well known that different parts of the skull grow at different rates and that the cranial base reaches its adult size first. The growth and development of the face and calvarium follows that of the basicranium, and growth of the basicranium is under tighter intrinsic control than either the face or the calvarium [14]. The basicranium has been argued to be the ‘central integrator’ of the entire skull, the module that links both the calvarium and the face [3,8]. We designed an experiment to test the hypothesis that perturbation of cartilage growth within synchondroses, a key developmental process that directly affects only the basicranium, via different genetic mechanisms will produce highly correlated changes in the shape of other skull units. We sought to demonstrate both the central role of the basicranium in determining adult skull shape, and the potential for the effect of a mutation affecting a single developmental process to propagate throughout a complex phenotype to produce similar, correlated morphological changes.

### Cartilage undergrowth produces similar craniofacial shapes across ontogeny, regardless of mechanism

Our results illustrate that inhibition of cartilage growth, either by inhibition of selenoprotein expression (UG1) or undersulfation of cartilage (UG2), produced a similar enchochondral phenotype that resulted in correlated, similar phenotypes in the rest of the skull. The UG1 model produced neonatal mice with posteriorly shortened basicrania, and correspondingly shortened faces and higher, more domed calvaria than their controls (Fig. 2). This phenotype was still present once the mice reached adulthood, and was also evident in our UG2 model, where a short, wide basicranium continued to be associated with a short, wide face and higher calvarium in comparison to the skulls of control mice (Fig. 3). These coordinated skull shape differences became increasingly pronounced as postnatal growth and development proceeded, resulting in an adult sample in which the UG models were clearly differentiated from the OG model and all controls along the first two axes of variation (Fig. 3).

Interestingly, we did not observe a similar (although predicted to be phenotypically opposite) effect in our OG model. Instead, these mice exhibited a relatively greater range of phenotypic variation, while clustering most closely with the control strains (both their own and those of the undergrowth models) in our analyses, an effect that is exaggerated later in ontogeny (Figs. 2, 3). While the *Pten* deletion present in these mice has been shown to produce endochondral bone overgrowth, it also seems to result in earlier closure of synchondroses due to cartilage cell hypertrophy [36]. Early and disordered closure of synchondroses could result in mice with greater relative variation in basicranial length, but whose basicrania do not continue to grow long enough to actually surpass the length of the basicrania of unaffected mice. Further study is required to verify these findings, and future work should include an attempt, possibly using a different model, to demonstrate the overgrowth effect we were searching for here.

While the shapes of both the face and the calvarium were found to be significantly correlated with the shape of the basicranium across ontogeny, our results demonstrate that these patterns are complex and change across postnatal development. The patterns of relationship among skull shapes described above are already evident at the neonatal stage, where a relatively shorter and wider basicranium is significantly associated with a shortened face (Fig. 4) and a more domed basicranium (Fig. 5). Although correlations between these modules were relatively high, they were not all significant (Table 4) and the percentage of covariation explained by the PLS axes is variable and only moderately high. We did not observe any obvious clustering along the PLS axes describing the relationship between the basicranium and either the calvarium or the face, but the slopes are similar for all groups indicating that the general pattern of covariance between parts of the skull is the same at this developmental stage (Figs. 4, 5). In the adult analysis, we recovered similar patterns of relationship among shapes, where once again short, broad basicrania were associated with short faces (Fig. 6) and broad, domed calvaria (Fig. 7). At this stage, correlations between modules were all highly significant, and the percentage of covariation explained increased drastically for the basicranium-calvarium relationship (Table 5). Interestingly, the first PLS axis between basicranium and calvarium (Fig. 7A), which explained more than 90% of the covariation, demonstrated substantial structuring according to treatment group. Both UG groups clustered together and exhibited similar slopes, reinforcing the notion that regardless of the means by which cartilage growth is inhibited, the results are both consistent and predictable. In contrast, the OG group exhibited two clusters at opposite ends of the axis, again reflecting the much greater range of variation present in this model and discussed above.

Our results also demonstrate that manipulations or mutations targeting chondrocranial growth, regardless of mechanism, have the effect of increasing the amount of phenotypic variation present in the sample in comparison to controls. This effect is evident in both neonatal (Figs. 4, 5) and adult (Figs. 6, 7) assessments of covariation patterns, where controls strains occupy a restricted region of morphospace while affected strains are scattered across the PLS axes. Despite this increase in variation, covariation patterns among skull parts do not change. This result provides further support for the presence of underlying developmental architecture acting to maintain coordination among functional units, despite the action of processes that are dramatically increasing the amount of variation produced [19,20,25].

Taken together, these results suggest that the effects of manipulations or mutations targeting a developmental process, while initiated very early on, develop their full effects over the course of ontogeny and become more and more pronounced as the organism proceeds toward the final, adult phenotype. Further, effects which may have an apparently minor effect when assessed relatively early in ontogeny will ultimately produce major variations in adult phenotype. This variation, however, is constrained to occur within an established underlying architecture that permits functional relationships to persist among developing parts of the phenotype. We note, however, that this study assessed only two time points, and the changes evident in these patterns of relationship require further investigation using a more complete ontogenetic series.

The results presented here should be interpreted in light of our decision to omit removal of the effects of allometry on shape. Allometry is often found to produce covariation among traits, thereby obscuring the often more subtle role of shape in generating such effects [46,50,51]. More recently, it has been argued that the effect of allometry on integration patterns should not be treated as a methodological nuisance, but rather as a biologically meaningful component of variation in evolutionary contexts [52]. We have chosen to retain allometric size in the present context, because our experimental design was predicated on direct manipulation of basicranial size through reduction in cartilage growth. The decision to retain allometric size also has consequences for the PLS analyses we employ. Here, we have used group-centered PLS in order to avoid the confounding effects of between- group variation, which may be of particular influence here because of the presence of allometric size in the dataset. A systematic comparison of the nature of integration patterns in the skull in the presence and absence of allometry is beyond the scope of the present study, but we caution that our results should be interpreted in light of our decision not to remove this information from our dataset, and we encourage further study of the possible role of allometric effects in structuring craniofacial development. We also note that Procrustes-based approaches such as those used here form part of a particular theory of shape that carries a set of assumptions. While a methodological comparison is beyond the scope of the present contribution, we encourage future work employing alternative approaches.

### Epigenetic interactions among developing parts of the skull and skull contents have an important role in structuring phenotypic variation

The patterns of phenotypic variation described here were generated by deliberately targeting cartilage growth within synchondroses, a developmental process that, by its nature, contributes directly only to the adult form of the endochondral bones comprising the basicranium, and not to any other skull elements included in this study. This experimental design therefore allowed us to measure both the *genetic* effects of inhibition of cartilage growth on the basicranium, and the *epigenetic* effects of inhibition of cartilage growth as they were transmitted through the basicranium to the face and calvarium.

We demonstrated that while inhibition of cartilage growth via at the synchondroses produced a predictably shortened basicranium, which we interpret as a direct, or genetic effect, it also resulted in an increase in basicranial width, evident in both the UG groups, which we interpret as an indirect, or epigenetic outcome, which is determined by basicranial shape and not by the mechanism itself. We further showed that this short, wide basicranium is associated with a shortened face and a globular calvarium, which we again interpret as epigenetic outcomes. The functional association between these three skull modules and the structures around them is key to our understanding of the skull as a unit and the means by which such interactions may generate shape change.

Arguably, housing the brain and supporting the sense organs and feeding apparatus are among the most important functions of the skull as a unit. The relationship between calvarium and brain is thought to be particularly robust, based primarily on size, and is known to persist throughout ontogeny [15]. While a series of complex molecular interactions are known to influence the developing brain and face [5,53,54], there are also important physical relationships between these elements, and several studies have demonstrated that reduction in brain size results in a shorter face [6,55]. While the effect of the Trsp mutation on brain size is unknown, the UG2 mice have been found to retain similar brain size compared to unaffected controls [42]. We propose that the phenotypic variation we have documented here is produced by epigenetic interactions among the developing skull modules, as well as between these modules and the developing brain. In order to house a brain of relatively normal size, the volume contained within the calvarium must remain unaffected by changes to the cranial base, and this is accomplished by the production of a more spherical shape. The calvarium and cranial base interact reciprocally to accommodate the unaffected brain. It is important to note that the relationship between the basicranium and the face is complicated by the effects of factors external to both, and is likely more nuanced than what we are able to show here. Growth hormone, in particular, has substantial effects on adult morphology and exerts its effects throughout ontogeny [16]. As the last part of the skull to reach adult size [14], the face is exposed to the effects of growth hormone for the longest period of time. Further work is required to disentangle the relationship between epigenetic and growth-hormone-mediated effects on adult facial morphology, but such work promises to provide exciting insight in to the mechanisms behind facial variation and dysmorphology in humans, and vertebrates more broadly.

It is possible that changes to skull shape might be driven by some mechanism other than endochondral growth. Both brain size and facial size, and their interrelationships, have been shown to be important for understanding hominin evolution [4,10], and changes to these structures implicate mechanisms that influence dermal bone growth and neurological development. The basicranium, however, reaches adult size before the calvarium and face [12,13], making it likely that mechanisms influencing endochondral bone growth are primary to those affecting dermal bone growth. It is possible that altered brain growth, and associated changes in sutural patterning and timing [15], might produce a phenotypic outcome similar to what we observed here, but considerable future work is required to determine if other mechanisms are able to produce a similar outcome.

The results reported here have interesting consequences for our understanding of primate, and in turn hominid, evolution. Past work examining the evolution of the hominid skull has tended to follow an adaptationist paradigm, as pointed out by Ackermann and Cheverud [56], with attempts being made to link skull features to diverse other putatively human traits such as bipedalism and the capacity for speech [7]. Fewer studies have taken the perspective that phenotypic diversity in modern human calvaria may be incidental to the evolutionary trajectory of the group [56–58]. Here, we propose that variation in the shape of the calvarium is substantially determined by epigenetic interactions with the basicranium and brain. It is conceivable that in this instance, calvarial shape is not directly adaptive, but rather is driven by selection on the basicranium instead. Our results further enhance our understanding of the key role that developmental architecture, in this case through mediation by epigenetic interactions, plays in structuring phenotypic variation. Future work, focusing on the nature of these interactions and the extent of their influence, will be key to elucidating the multifaceted evolutionary history of the hominid skull and other, equally complex, morphological features.

We have shown that inhibition of cartilage growth within synchondroses via different genetic mechanisms produces similar phenotypic changes in the basicranium. These changes are accompanied by highly correlated changes in the calvarium and face, indicating that important phenotypic effects can be generated at a distance from the actual locus of natural selection as it acts upon a single phenotypic element. Further, we have demonstrated that patterns of integration among skull modules become more densely structured while retaining similar features over ontogeny, developing stronger effects, within the bounds of architectural constraints, as final adult phenotypes take shape. Finally, we have demonstrated a key role for epigenetic interactions among developing skull elements and the brain in the production of substantial, yet constrained, phenotypic variation. These results reinforce the notion that evolvability is enhanced by developmental architecture, and that understanding the role of developmental architecture in the production of phenotypic variation is key to understand the evolutionary history of organisms. This work is of particular importance for our understanding of hominid evolution, where focus on skull morphology has informed a long tradition of adaptationist thought that may benefit from a novel approach.
